# Environment of origin and domestication affect morphological, physiological, and agronomic response to water deficit in chile pepper (*Capsicum* sp.)

**DOI:** 10.1371/journal.pone.0260684

**Published:** 2022-06-14

**Authors:** Jack E. McCoy, Leah K. McHale, Michael Kantar, Lev Jardón-Barbolla, Kristin L. Mercer

**Affiliations:** 1 Department of Horticulture and Crop Science, Ohio State University, Columbus, OH, United States of America; 2 Department of Tropical Plant and Soil Sciences, University of Hawai’i, Manoa, Honolulu, HI, United States of America; 3 Centro de Investigaciones Interdisciplinarias en Ciencias y Humanidades, Universidad Nacional Autónoma de México, Mexico City, Mexico; KGUT: Graduate University of Advanced Technology, ISLAMIC REPUBLIC OF IRAN

## Abstract

Global climate change is having a significant effect on agriculture by causing greater precipitation variability and an increased risk of drought. To mitigate these effects, it is important to identify specific traits, adaptations, and germplasm that improve tolerance to soil water deficit. Local varieties, known as landraces, have undergone generations of farmer-mediated selection and can serve as sources of variation, specifically for tolerance to abiotic stress. Landraces can possess local adaptations, where accessions adapted to a particular environment will outperform others grown under the same conditions. We explore adaptations to water deficit in chile pepper landraces from across an environmental gradient in Mexico, a center of crop domestication and diversity, as well in improved varieties bred for the US. In the present study, we evaluated 25 US and Mexico accessions in a greenhouse experiment under well-watered and water deficit conditions and measured morphological, physiological, and agronomic traits. Accession and irrigation regime influenced plant biomass and height, while branching, CO_2_ assimilation, and fruit weight were all influenced by an interaction between accession and irrigation. *A priori* group contrasts revealed possible adaptations to water deficit for branching, CO_2_ assimilation, and plant height associated with geographic origin, domestication level, and pepper species. Additionally, within the Mexican landraces, the number of primary branches had a strong relationship with precipitation from the environment of origin. This work provides insight into chile pepper response to water deficit and adaptation to drought and identifies possibly tolerant germplasm.

## Introduction

Global temperatures are rising due to anthropogenic climate change, causing greater precipitation variability and an accelerated risk of drought [1]. Increasing incidence of drought has considerable implications for agriculture. Crop genetic improvement, such as breeding of varieties capable of tolerating drought, could mitigate the effects of climate change on agriculture [2]. In order to do this, we must first identify sources of diversity and specific traits associated with drought adaptation and water deficit tolerance. Landraces, i.e., local crop varieties from centers of diversity, have undergone less intentional breeding, thus representing a different level of domestication than commercial cultivars and may serve as sources for biotic and abiotic stress tolerance [3–5]. Yet, there is also growing evidence that substantial diversity can be maintained under conventional breeding, as seen recently in tomato [6]. Still, landraces likely possess higher variation for response to abiotic stress and require further exploration, alongside their more commercial counterparts. Additionally, this understanding of variation between commercial and landrace germplasm could converge to inform participatory plant breeding programs aimed at addressing local farmers needs for climate change adaptation [7, 8].

Chile pepper (*Capsicum* sp.) is an economically and culturally significant crop worldwide, with over 36 million tons harvested in 2017 [9]. Mexico is the second largest producer and the center of domestication and diversity for chile pepper [9–11]. Near the center of domestication and diversity, crops and their wild relatives may possess adaptations to local conditions [12]. These adaptations may result from genetic differentiation for traits affecting productivity, such as the phenotypic responses to varied growing environments [13]. For example, in their local environments, Mexican maize (*Zea mays* L. subsp. *mays*) landraces from a given elevation tend to demonstrate higher overall fitness than non-local ones; local landraces also tend to outperform improved varieties under local conditions [14]. In wild and landrace chile pepper, local adaptation may also be possible, where individuals (referred to as accessions) originating from low precipitation environments may possess elevated tolerance to soil water deficit. For example, a recent germination study identified adaptive responses in chile pepper associated with precipitation [15]. Landraces from hotter, drier ecozones had significantly delayed germination, suggesting a possible adaptive response related to drought avoidance. Evidence of significant genetic diversity exists in modern pepper germplasm in the US, particularly for biotic stress tolerance [e.g. 16, 17]; however, landrace germplasm likely possesses more genetic variation. Phenotypic diversity can also differ greatly between landrace and commercial chile peppers including differences in fruit type and overall plant architecture, which can both affect productivity, e.g., with greater branching providing more meristematic regions for fruit production. Yet, it is unclear how this diversity differs across environments or how it contributes to response to water deficit. For this reason, it is important to explore tolerance to soil water deficit in both US and Mexican accessions, including plant architectural and physiological responses.

This greenhouse study evaluated the response to soil water deficit in a diverse set of germplasm selected from US and Mexican origins. The objectives of this experiment were to: (1) measure the morphological, physiological, and agronomic response of diverse chile pepper to soil water deficit; (2) evaluate differences between accessions, including differences associated with environmental and geographic origin and domestication gradients; and (3) identify unique responses of accessions to soil water deficit (i.e., genotype by environment interactions) that may indicate a tolerance to soil water deficit. Results of this study offer insight into adaptations to drought in chile pepper and highlight germplasm for continued research in water deficit tolerance.

## Materials and methods

### Plant material

Our 25 chile pepper accessions included 18 from the US and seven from Mexico (Table 1). U.S. accessions were no longer under USDA plant variety protection status and had diverse plant habit, fruit type, and origin (Table 1). Specific US accessions included one commercial bell, one cherry, one *chilhuacle* landrace from Mexico that is produced in and selected for the Northwestern US, three commercial *jalapeño* cultivars, six New Mexican cultivars or landraces, one ornamental, two paprika (one US bred and one bred in Hungary, both produced in the US), one Mexican *serrano* type that is produced in the US, and two sweet peppers.

**Table 1:** List of germplasm used in a greenhouse soil water deficit experiment on chile pepper at the Ohio State University. Accessions are organized by country of origin.

Name^a^	Species^b^	Pod Type	Seed Source	Origin Description
U.S. Germplasm				
Kaala	*C*. *annuum*	Bell	UHM Seed Lab	Developed and produced by University of Hawaii
PI 592813	*C*. *annuum*	Cherry	NPGS	Maintained by the plant Genetic Resources Conservation Unit, Georgia
Chilhuacle Negro	*C*. *annuum*	*Chilhuacle*	Adaptive Seeds	Mexican landrace, produced in Oregon
Waialua	*C*. *annuum*	Jalapeño	UHM Seed Lab	Developed and produced by University of Hawaii
Tam Jalapeño	*C*. *annuum*	Jalapeño	Reimer Seeds	Developed by Texas A&M University, produced in Maryland
Tam Vera Cruz	*C*. *annuum*	Jalapeño	Reimer Seeds	Developed by Texas A&M University, produced in Maryland
Canoncito	*C*. *annuum*	New mexican	Wild Garden Seed	New Mexican landrace, produced in Oregon
Chimayo	*C*. *annuum*	New mexican	Adaptive Seeds	New Mexican landrace, produced in Oregon
Anaheim M	*C*. *annuum*	New mexican	Reimer Seeds	U.S. cultivar, produced in Maryland
Anaheim TMR 23	*C*. *annuum*	New mexican	Reimer Seeds	U.S. cultivar, produced in Maryland
PI 586666	*C*. *annuum*	New mexican	NPGS	Maintained by the plant Genetic Resources Conservation Unit, Georgia
PI 159229	*C*. *annuum*	New mexican	NPGS	Maintained by the plant Genetic Resources Conservation Unit, Georgia
PI 631153	*C*. *annuum*	Ornamental	NPGS	Maintained by the plant Genetic Resources Conservation Unit, Georgia
Szegedi 179	*C*. *annuum*	Paprika	Adaptive Seeds	Originated in Hungary, produced in Oregon
NuMex Conquistador	*C*. *annuum*	Paprika	Reimer Seeds	Developed by New Mexico State University, produced in Maryland
Hildalgo Hot	*C*. *annuum*	Serrano	Reimer Seeds	Originated in Mexico, produced in Maryland
Stocky Golden Roaster	*C*. *annuum*	Sweet	Wild Garden Seed	Farm-original variety, Oregon
PI 586665	*C*. *annuum*	Sweet	NPGS	Maintained by the plant Genetic Resources Conservation Unit, Georgia
Mexico Germplasm				
Ca0057	*C*. *annuum*	*Chile de agua*	OSU collection	Collected from Oaxaca central valley plantation
Ca0256	*C*. *annuum*	*Chilgole*	OSU collection	Collected from Oaxaca coast backyard
Ca0045	*C*. *annuum*	*Costeño rojo*	OSU collection	Collected from Oaxaca coast plantation
Ca0310	*C*. *annuum*	*Tusta*	OSU collection	Collected from Oaxaca central valley letstand
Ca0344	*C*. *annuum*	*Tusta*	OSU collection	Collected from Oaxaca central valley letstand
Cc0144	*C*. *chinense*	*Habanero*	OSU collection	Collected from Oaxaca coast backyard
Cf0173	*C*. *frutescens*	*Mirasol*	OSU collection	Collected from Oaxaca coast backyard

^a^Names refers to given cultivar, landrace name, or database accession number used for USDA and Mexico germplasm.

^b^Genus for all species is *Capsicum*.

We chose the seven Mexican accessions from an array of chile peppers collected from diverse environments in the Mexican state of Oaxaca as potential sources of tolerance to water deficit based on a combination of their environment of origin, performance in a prior greenhouse study, and results from an environmental association analysis that used environment of origin as a “phenotype” [18]. In addition to their possible drought adaptations, the seven accessions serve as parents in on-going population development for continued genetic studies. Mexican accessions included five *C*. *annuum*–one *chile de agua* from a central valley plantation, one *chilgole* from a costal backyard, one *costeño rojo* from a costal plantation, and two *tusta* semi-wild from the central valley–and two additional species. The *C*. *chinense* and *C*. *frutescens* accessions were a *habanero* and *mirasol*, respectively, and had been collected from costal backyards. Accessions were collected from both private and public land with direct consent from the landowner or community representative. This work did not involve endangered or protected species.

### Study system and experimental design

We grew the 25 chile pepper accessions under greenhouse conditions at the Ohio State University in Columbus, Ohio. Seedlings were transplanted into 6 L pots using BM-1 Potting Mix (Berger, Saint-Modeste, QC, Canada) approximately six weeks after planting. Slow release, 14-14-14 fertilizer (Osmocote, Scotts Miracle-Gro, Marysville, OH, USA) was mixed into the soil at transplanting and three fertilizer applications of a 20-10-20 Peat-Lite fertilizer (JR Peters Inc., Allentown, PA, USA) were applied through irrigation lines, as needed.

We used a randomized complete block design with three blocks, individual greenhouse bench served as our blocking factor. Within each block we applied factorial combinations of accession (25 levels, see above) and irrigation (water deficit and control, see below) to each pot (one plant per pot) or experimental unit.

Plants were subjected to two levels of irrigation: daily watering (control) and weekly watering (water deficit). Water application varied slightly throughout the growing season to accommodate light, temperature, and transpiration rate changes; however, plants were watered to saturation at each watering event. Stomatal conductance (Gsw) and CO_2_ assimilation (Anet) were measured using the LI-6800 Portable Photosynthesis System (Licor, Lincoln, NE, USA). Survey-style measurements were collected on a healthy, fully expanded leaf in the middle of the canopy between 10 am and 12 pm. Instrument conditions were as follows: Flow rate 600 μmol/s, relative humidity 65%, CO_2__s 400 ppm, fan speed 10,000 rpm, and the light source was set to ambient light conditions. Prior to logging a measurement, the LI-6800 was stabilized on relative humidity, Gsw, and Anet. After approximately four months, plants were destructively harvested and measurements of morphological and agronomic traits were collected, including: plant height (PH), above-ground plant biomass (PBi), number of primary branches (PBr), fruit weight (FW), and 100-seed weight (SW). Due to the varying phenology within Mexican accessions, many accessions were still fruiting as the experiment was terminated. Thus, we only report on analyses of fruit traits for US accessions.

### Data analysis

To perform three different sets of analyses, we used the lmerTest package in R version 4.0.5 [19]. First, to assess the effect of experimental treatments (fixed and random) on our measured traits, we employed a general linear mixed model for analysis of variance (ANOVA). For growth, morphological, and physiological traits, we analyzed data from all accessions with irrigation, accession, and their interaction as fixed effects, and block as a random effect. Due to limitations on our fruit data in Mexican accessions (see above), we analyzed just the 18 U.S. cultivars to assess agronomic performance (FW, SW). For all analyses, ANOVA assumptions were checked (identification of outliers, normal distribution of residuals, equality of variance, and independence of error). Log transformations were performed on Anet and Gsw due the heteroscedastic nature of the data. For significant effects, we generated mean separation tables using Tukey’s HSD test.

Second, to test effects of country of origin, landrace status, and *Capsicum* species, we conducted a series of *a priori* contrasts within the previous model. To measure the effect of country of origin, we contrasted Mexican and U.S. germplasm. We opted to contrast landrace status because several U.S. accessions in this experiment are marketed as either U.S. or Mexican landraces. Though not collected directly from Mexico, it is possible that these accessions may have undergone less commercial breeding and subsequently possess adaptations to abiotic stress [5]. Thus, we contrasted landraces (both US and Mexico) with others. Since plant habit can differ greatly across species, we contrasted our *C*. *chinense* (Cc0144) and our *C*. *fructescens* (Cf0173) accessions with our *C*. *annuum* ones (Table 1) to better understand differences due to speciation.

Third, to address the relationship between environment of origin and response to water deficit in our collected traits, we performed regression analysis using only Mexican accessions. Our collection of Mexican accessions are georeferenced and thus can be analyzed for specific environmental variables from their originating location using the publicly available Bioclim data and soil data from the ISRIC [20, 21]. We selected variables directly related to precipitation, including total available soil water capacity, BIO12 (annual mean precipitation), BIO15 (precipitation seasonality), BIO16 (precipitation of the wettest quarter), BIO17 (precipitation of the driest quarter), BIO18 (precipitation of the warmest quarter), and BIO19 (precipitation of the coldest quarter). In order to reduce the overall size of the linear model, we generated a correlation matrix of the seven environmental variables (S1 Fig). Using the corresponding P values and correlation coefficients (S1 Fig and S1 Table), we identified all significant correlations (α = 0.05, coefficient > 0.80) and determined that a linear model using total available soil water, annual mean precipitation, and precipitation seasonality would be representative of the full model. The experimental blocking factor contributed very little to variation in the model and was confirmed to be unnecessary in all models based on lower Akaike information criterion (AIC) scores for models without block. Ultimately, we used a linear regression model including the three environmental variables (total available soil water, annual mean precipitation, and precipitation seasonality) plus irrigation treatment and its interaction with each variable.

## Results

### Response to water deficit, accession and their interactions

Results indicated that PBi and PH were both significantly reduced by water deficit by an average of 10.6 g (16.3%) and 7.6 cm (12.3%), respectively (Tables 2 and S2). Mean PBi and PH were also significantly affected by accession (Tables 2 and 3). The PBi means ranged significantly among accessions from 49.6±3.30 g (Szegedi 179) to 75.7±3.30 g (Ca0344); PH ranged from 36.2±4.30 cm (PI631153) to 81.2±4.30 cm (Chilhuacle Negro) (Table 3).

**Table 2:** Analysis of variance for seven traits in a greenhouse soil water deficit experiment on chile pepper (*Capsicum* sp.) at the Ohio State University. Data was analyzed in R (version 4.0.5) using a linear mixed model. Source was considered significant if P < 0.05.

Trait	Source	DF	F Value	P^a^
U.S. and Mexico Germplasm^b^				
Primary Branching	Accession	24	30.20	***
	Irrigation	1	12.57	***
	Accession by Irrigation	24	2.40	**
CO_2_ Assimilation^c^	Accession	24	3.53	***
	Irrigation	1	0.03	NS
	Accession by Irrigation	24	1.89	*
Stomatal Conductance^c^	Accession	24	1.59	NS
	Irrigation	1	0.00	NS
	Accession by Irrigation	24	1.41	NS
Plant Biomass (no fruit)	Accession	24	2.31	**
	Irrigation	1	64.87	***
	Accession by Irrigation	24	0.88	NS
Plant Height	Accession	24	8.46	***
	Irrigation	1	23.70	***
	Accession by Irrigation	24	1.29	NS
U.S. Germplasm				
Total Fruit Weight	Accession	17	11.03	***
	Irrigation	1	119.24	***
	Accession by Irrigation	17	3.51	***
100-Seed Weight	Accession	17	1.11	NS
	Irrigation	1	0.00	NS
	Accession by Irrigation	17	1.08	NS

^a^*, **, ***specify significant differences at P values of 0.05, 0.01, and 0.001 respectively.

^b^Yield traits (total fruit weight and 100-seed weight) were analyzed only in U.S. germplasm because of variable phenology between U.S. and Mexican accessions.

^c^Log transformation performed to account for heteroscedastic data distribution.

**Table 3:** Mean separation (estimated marginal means) of two traits with significant main affects for accession from a greenhouse soil water deficit experiment on chile pepper (*Capsicum* sp.) at the Ohio State University. Organized alphabetically within country of origin.

Accession	Plant biomass (g)^a^		SE^b^	Plant height (cm)^a^		SE
U.S. Germplasm						
Anaheim M	59.0	abc	3.30	67.8	abcde	4.30
Anaheim TMR23	56.8	bc	3.30	43.2	fg	4.30
Canoncito	53.8	bc	3.30	60.8	abcdef	4.30
Chilhuacle Negro	64.4	abc	3.30	81.2	a	4.30
Chimayo	61.2	abc	3.30	68.7	abcd	4.30
Hidalgo Hot	62.6	abc	3.30	69.2	abc	4.30
Kaala	67.5	ab	3.30	49.0	cdefg	4.30
NuMex Conquistador	56.0	bc	3.30	49.7	bcdefg	4.30
PI159229	60.4	abc	3.30	55.3	bcdefg	4.30
PI586665	56.8	bc	3.30	68.3	abcd	4.30
PI586666	59.3	abc	3.30	70.2	ab	4.30
PI592813	59.4	abc	3.30	69.0	abc	4.30
PI631153	58.7	abc	3.30	36.2	g	4.30
Stocky Golden Roaster	58.9	abc	3.30	62.3	abcdef	4.30
Szegedi 179	49.6	c	3.30	61.3	abcdef	4.30
Tam Jalapeno	58.1	bc	3.30	49.7	bcdefg	4.30
Tam Vera Cruz	59.7	abc	3.30	48.7	cdefg	4.30
Waialua	62.4	abc	3.30	52.7	bcdefg	4.30
Mexico Germplasm						
Ca0045	56.0	bc	3.30	63.2	abcdef	4.30
Ca0057	61.4	abc	3.30	65.7	abcde	4.30
Ca0256	56.8	bc	3.30	48.0	defg	4.30
Ca0310	57.9	bc	3.30	39.2	g	4.30
Ca0344	75.7	a	3.30	64.3	abcde	4.30
Cc0144	59.4	abc	3.30	47.2	efg	4.30
Cf0173	52.9	bc	3.30	49.3	cdefg	4.30

^a^Different letters indicate significant differences by Tukey’s HSD Test (P = 0.05) between accessions.

^b^Indicates standard error of the mean.

**Fig 1:**
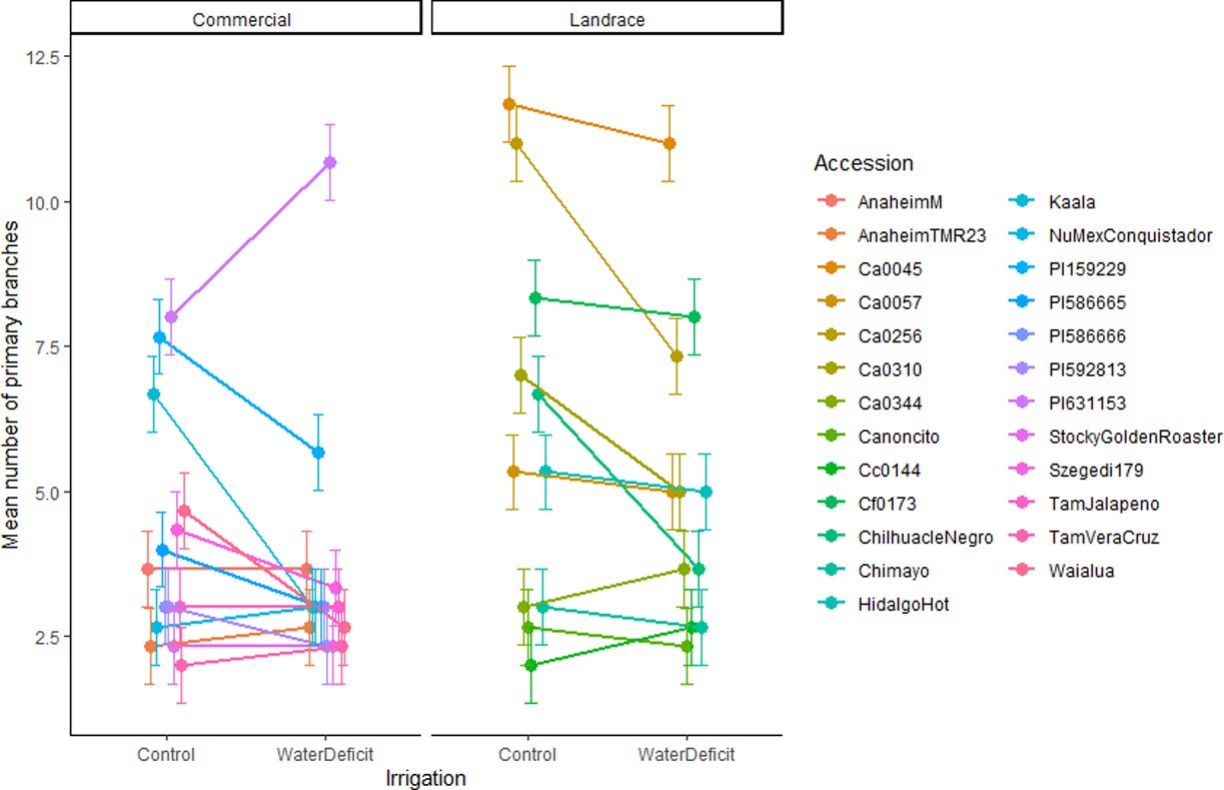
Interaction of irrigation and accession means with standard error bars for primary branch number in a greenhouse soil water deficit experiment on chile pepper (*Capsicum* sp.) at the Ohio State University. Accessions are separated by domestication level.

**Fig 2:**
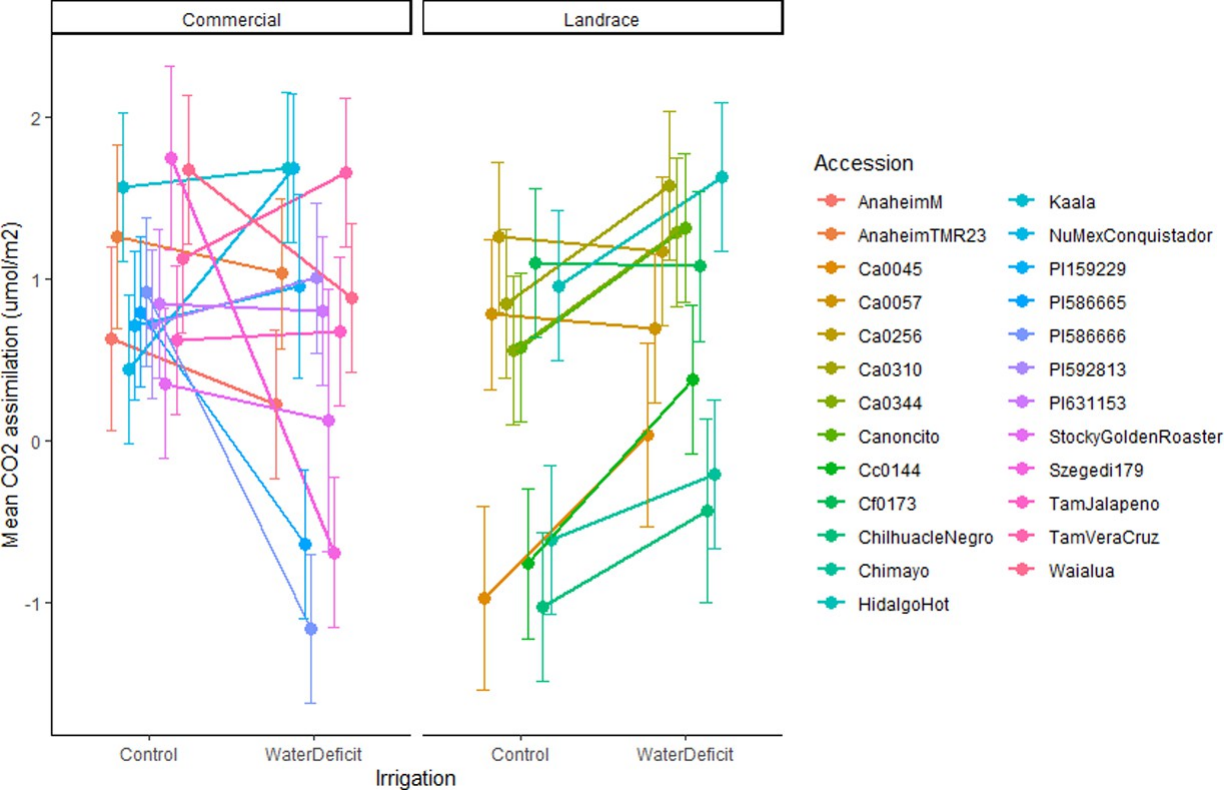
Interaction plot of estimated marginal means with standard error bars for CO_2_ assimilation (log transformed) in a greenhouse soil water deficit experiment on chile pepper (*Capsicum* sp.) at the Ohio State University. Accessions are separated by domestication level.

Although PBr and Anet were both influenced by the main effect of irrigation and/or accession, a significant interaction between accession and irrigation indicated a differential response to water deficit among accessions (Tables 2 and S3). Many accessions experienced decreased PBr under water deficit, but to differing degrees (Fig 1) and some accessions experienced no change in PBr. Surprisingly, the USDA accession PI631153 had a great increase in PBr under water deficit. Anet rates in commercial US accessions showed a range of responses (i.e., they declined, stayed the same, or increased) under water deficit (Fig 2). However, Anet rates in landrace accessions (from US and Mexico) either remained the same or increased under water deficit but did not decline.

**Fig 3:**
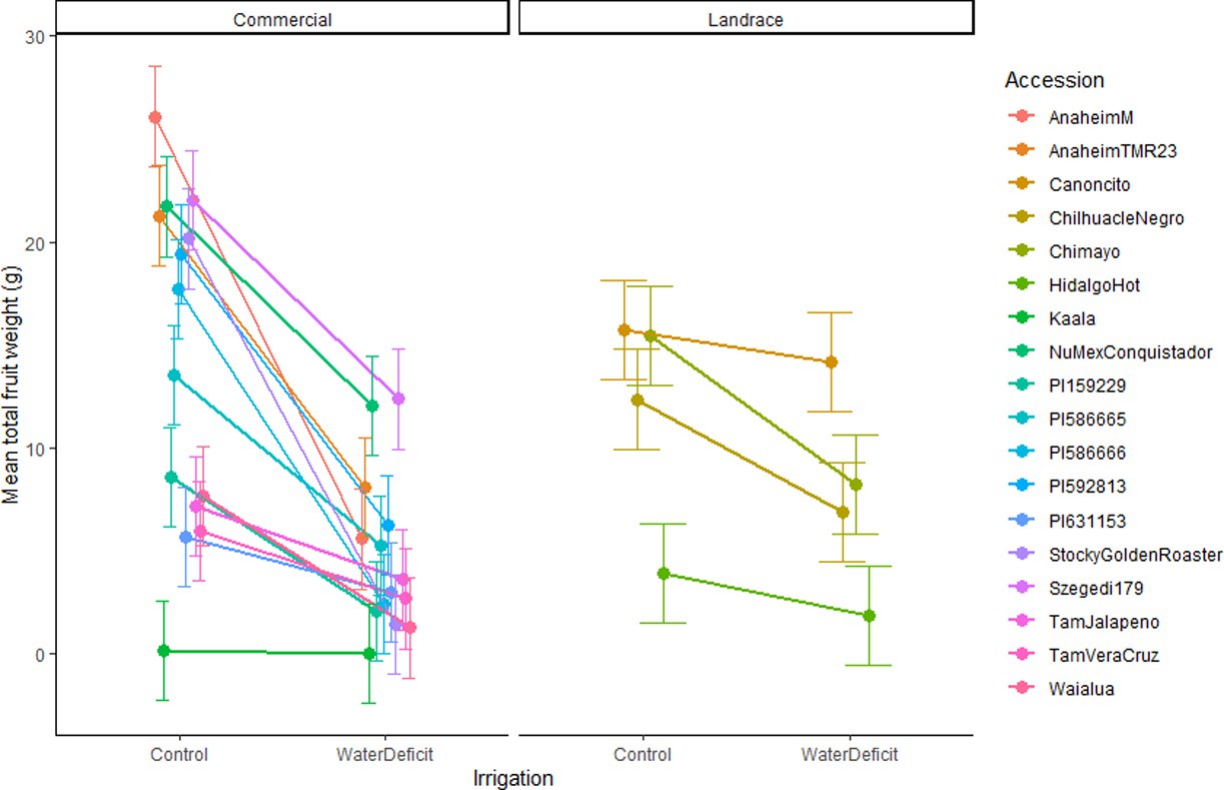
Interaction plot of estimated marginal means with standard error bars for mean fruit weight in U.S. accessions only in a greenhouse soil water deficit experiment on chile pepper (*Capsicum* sp.) at the Ohio State University. Accessions are separated by domestication level.

Focusing only on US accessions for agronomic traits, we found a significant variation in yield response to water deficit among accessions (Table 2 and Fig 3). In general, FW was reduced under water deficit, but to varying degrees. Anaheim M had the greatest total FW under well-watered conditions but had a substantial decrease under water deficit. This pattern of higher yielding varieties having the greatest decline was also true for Anaheim TMR23, Stocky Golden Roaster, PI586666 and PI592813, but less so for Szegedi 179 and NuMex Conquistador, which were among those with the highest FW under both control and water deficit. By contrast, Canoncito, the highest yielding landrace, declined little in total FW with water deficit, suggesting possible tolerance (Fig 3).

### Environment of origin and domestication effects

Examining our *a priori* contrasts, we found significant differences between groups Mexico vs. US, Landrace vs. other, and Annuum vs. other (Table 4). Under both well-watered and water deficit conditions, PBr was significantly higher in Mexican accessions than US accessions and in landraces as compared to improved varieties (Table 4). The PBr was significantly lower in *C*. *annuum* than in other species under water deficit, but not under well-watered conditions (Table 4). Plants from the US were taller than those from Mexico under well-watered conditions, but we found no differences between the two under water deficit (Table 4). Additionally, *C*. *annuum* accessions were taller than accessions from other species under well-watered conditions, but that difference declined under water deficit (Table 4).

**Table 4:** Results of *a priori* group contrasts on four traits from a greenhouse soil water deficit experiment on chile pepper (*Capsicum* sp.) at the Ohio State University.

Trait	Mexico:US^a^				Landrace:Other				Annuum:Other			
	Control		Water Deficit		Control		Water Deficit		Control		Water Deficit	
	t value	P^b^	t value	P	t value	P	t value	P	t value	P	t value	P
Primary Branching	9.44	*** >	8.69	*** >	7.26	*** >	5.72	*** >	-0.53	NS	-2.38	* <
CO_2_ Assimilation	-1.58	NS	1.40	NS	-3.69	*** <	0.95	NS	1.52	NS	-0.18	NS
Plant Biomass	1.62	NS	-0.77	NS	1.88	NS	-0.34	NS	0.24	NS	1.78	NS
Plant Height	-2.38	* <	-1.88	NS	1.87	NS	1.5	NS	3.05	** >	1.95	NS

^a^Contrasts were made in the following three groups: Mexico vs. U.S. accessions, Landrace accessions vs. others, and *C*. *annuum* vs. others.

^b^*, **, ***specify significant differences at P values of 0.05, 0.01, and 0.001 respectively. Greater than/less than symbols indicate the direction of the contrast vis-à-vis the order of the groups in the heading.

CO_2_ assimilation was significantly lower in landraces than in other accessions when well-watered. Yet, we found no differences under water deficit (Table 4). This reinforces the previously mentioned significant interactions between accession and irrigation on Anet, indicating that assimilation rates were maintained within landrace accessions across irrigation treatments, but not in US accessions.

Lastly, results of the multiple regression analysis suggest that key environmental parameters related to precipitation are significant predictors of PBr (Table 5). More specifically, we found a significant negative linear relationship between PBr and total available soil water and precipitation seasonality (Table 5). Each unit increase in soil water content predicted a decrease in 2.699 PBr (SE = 0.430, P ≤ 0.001) and each unit increase in precipitation seasonality predicted a decrease in 0.214 PBr (SE = 0.073, P = 0.006). We identified a significantly positive relationship between annual mean precipitation and PBr, where each unit of increase in precipitation predicts an increase in 0.014 PBr (SE = 0.002, P ≤ 0.001). Contrary to results of the ANOVA, irrigation treatment did not have a significant relationship with PBr. This suggests that environment plays a more significant role than water regime in predicting PBr. Overall, the model accounted for 69.7% of the variation in the data (Table 5).

**Table 5:** Multivariate regression analysis of Mexican chile pepper accessions from a greenhouse soil water deficit experiment on chile pepper (*Capsicum* sp.) at the Ohio State University. The relationship between mean primary branching and environmental parameters related to precipitation from the originating environment are presented (R^2^ = 0.697).

Trait	Predictors	Beta^a^	SE^b^	P^c^
Primary Branching	Irrigation	-14.803	33.451	NS
	Total Available Soil Water	-2.699	0.430	***
	Annual Mean Precipitation	0.014	0.002	***
	Precipitation Seasonality	-0.214	0.073	**
	Irrigation by Total Available Soil Water	0.328	0.609	NS
	Irrigation by Annual Mean Precipitation	-0.003	0.003	NS
	Irrigation by Precipitation Seasonality	0.023	0.103	NS

^a^Beta estimate indicates the change in primary branching relative to one unit change of the predictor.

^b^Indicates standard error of the mean.

^c^*, **, ***specify significant relationship at P values of 0.05, 0.01, and 0.001 respectively.

## Discussion

We found clear variation in responses of chile pepper accessions to soil water deficit, confirming that variation in tolerance may exist in germplasm currently in use. Much of the plant response to water deficit pointed to clear reductions in growth, but PBr and Anet did not respond consistently to water deficit across accessions. In fact, Mexican accessions, and landraces overall, better maintained Anet under water deficit than their improved counterparts. Moreover, Mexican accessions branched more than US accessions, landraces were branchier than improved accessions, and *C*. *annuum* branched less than other species when well-watered. Finally, we found precipitation of the originating environment to predict PBr in Mexican landraces. Thus, variation in physiological and growth form attributes in Mexican pepper landraces may be a consequence of selection for adaptation to specific precipitation regimes.

### Influence of water deficit on plant architecture and physiology

The wide range of PBr within our accessions and the varying response of PBr to water deficit suggests that plant architecture may play an important role in adaptation to drought. Branching may have biological significance relative to response to soil water deficit through canopy management for optimal transpiration rates and shoot to root biomass partitioning [e.g., 22, 23]. However, PBr likely plays a specific role in water deficit response that goes beyond biomass partitioning, as the effect of water deficit on above-ground biomass did not vary by accession. Leaf area index (LAI), the ratio of leaf area to ground area, plays a significant role in plant photosynthesis and generally decreases under drought [24]. Drought stress not only reduces leaf area, but also the photosynthetic rate per unit leaf area [23]. Shifts in plant architecture through additional PBr may optimize overall leaf area, thereby counteracting some of the reduced area caused by water stress. In addition to leaf area management, increased PBr may help reduce photosynthetic stress brought on by drought by providing a physical barrier that decreases direct light penetrating the canopy, reducing excessive photosynthetic energy. Branching undoubtedly influences adaptation to water deficit, but without additional data on leaf area, it is difficult to fully explain its role here.

In chile pepper, the influence of water deficit on PBr is variable and dependent on the germplasm. Showemimo and Olarewaju [25] found that PBr in an advanced pepper breeding line decreased with severity of drought applied whereas we saw inconsistent responses across our diverse germplasm. However, this reduction in PBr with breeding is also reflected in our data where improved, US accessions had lower PBr. Bernau [18], using landraces from southern Mexico, indicated that PBr may relate to environmental adaptation. In her work, PBr was lower in chile pepper accessions from the wet, western coast of Oaxaca and higher in accessions from the drier ecozones, Eastern Oaxaca and the Yucatan peninsula, reinforcing our results. Interestingly, Bernau also found PBr decreased in accessions from more intensively cultivated systems (i.e. was higher in naturally occurring forest accessions and lower in accessions from managed plantations), further emphasizing that domestication and improvement may have selected for reduced PBr despite the more open plantation environment on which crops might benefit from higher PBr. This change in plant architecture and subsequent reduction in branching associated with domestication has also been well-studied in maize, for example [26]. Perhaps landraces cultivated in plantation or milpa environments gain an environmental resilience from the increased PBr that their more heavily bred counterparts lack. Outside of pepper, morphological plasticity in response to water deficit has been shown to differ across levels of domestication. In seven cultivated and wild pairs, norms of reaction differed in that domesticated accessions greatly decreased plant biomass and growth rate in response to water deficit, but their wild progenitors had a less severe response [27]. Differing PBr responses to water deficit by US and Mexican accessions from across a domestication gradient require further investigation.

Similar to PBr, the differential Anet responses we found across accessions indicate possible physiological adaptations to drought. The general expectation of plants under water stress is that Anet rates decreases due to declines in Gsw to conserve water. We saw reductions in Anet under water deficit here in some commercial varieties, but, intriguingly, not in our landraces, while Gsw did not respond to irrigation. The Anet and Gsw data collected from one leaf at one time cannot explain whole-plant dynamics or responses over the course of the life cycle, especially since the relationships between Anet, Gsw, and whole-plant photosynthetic activity are tightly connected and complex [28, 29]. Leaf area and distribution, in addition to Anet per unit area, could be a significant factor for understanding plant growth under stress [29]. In fact, changes in plant architecture, leaf area, and specific leaf area may influence water use efficiency (WUE) and whole-plant gas exchange, as can stomatal density and size [30]. Thus, further studies of water deficit tolerance in chile pepper should explore more mechanistically the ways that physiological traits can combine to influence whole-plant responses.

### Influence of environment of origin and domestication on plant response to water deficit

We observed a geographic (i.e. Mexico vs. US) and domestication (i.e. landrace vs. other) effect on plant architecture, where PBr was highest in Mexican accessions and landraces (both from Mexico and the US) across irrigation treatments (Table 4). Interestingly, we found that some accessions, particularly landraces, either maintained or even increased Anet under water deficit (Fig 2). Lower Anet rates observed in landraces under well-watered conditions, when compared with improved accessions, suggests a couple of possibilities. First, improved accessions likely undergo a more typical response to water stress, i.e. an overall reduction in photosynthetic activity. Second, landrace accessions have a lower photosynthetic capacity under ideal conditions but demonstrate more resilience to stress by maintaining similar rates of assimilation compared with their improved counterparts. This may be an example of differential plasticity between landraces and more improved accessions. For example, in other productivity traits, improved accessions are more productive under well-watered conditions, but experience a greater loss in response to water deficit than landraces, as seen in several species when compared to their wild counterpart [27]. It is also possible that level of domestication and breeding have contributed to morphological changes, which in-turn affect whole plant physiology. Examples of these morphological changes were reported by Milla and Matesanz [31], where domesticated plants, when compared with their wild counterparts, invested more resources into leaf production than stem production, while maintaining similar photosynthetic rates at the individual leaf. It may also be important to consider cultivation systems associated with landrace chile pepper. For example, landraces are frequently found in milpa polyculture systems, where multiple crop species are grown simultaneously, some of which may be perennial. Increased PBr could be favorable for improved light capture and microclimate management when competition for resources may be present. However, additional study with emphasis on cultivation system is required to properly address this hypothesis. Finally, the relationship between precipitation parameters from the originating environment and PBr variation in Mexican accessions further suggests that PBr may be an adaptation associated with water availability (Table 5). Morphological adaptations associated with environment of origin have been identified in other studies, for example with stomatal size and density in Arabidopsis [30].

### Evidence of water deficit tolerant germplasm

We have identified traits in chile pepper that may provide adaptation to water deficit, such as PBr and Anet, which warrant further study. Some accessions expressed values of these traits that might make them relatively more water deficit tolerant or susceptible. For instance, the Mexican accession Ca0344 stands out as having exceptionally high productivity (measured as plant above-ground biomass), despite water deficit (and maintains steady PBr and Anet rates when water deprived. On the other end of the spectrum, US variety PI586666 (New Mexican type), had lower biomass overall and had reduced Anet rates under water deficit. US accessions maintaining yield across irrigation treatments may be classified, for agronomic purposes, as water deficit tolerant. Most notable in this experiment was the New Mexico landrace, Canoncito, which has relatively high FW, maintains FW even when water deprived and could be an interesting source of germplasm for breeding drought tolerance in the US. Canoncito, as well as Szegedi 179 and NuMex Conquistador, would be considered to be expressing a water deficit tolerant response, particularly when compared with a very susceptible accession, such as Anaheim M, whose high fruit production plummeted under water deficit.

## Conclusion

With increasing risk of drought to agriculture, tolerance to soil water deficit is of the utmost importance. This study demonstrated differential response of above-ground traits to soil water deficit in diverse chile pepper germplasm selected from the U.S. and Mexico. Results provide opportunity for continued study in drought adaptation and soil water deficit tolerance in chile pepper, specifically through the identification of unique morphological and physiological responses to water deficit and high performing accessions.

## Supporting information

S1 Fig Correlation matrix for seven environmental variables associated with origin of Mexican chile pepper landraces. Size and color indicate strength of the corelation (larger and darger blue is more positively correlated and larger and darger red is more negatively correlated). Variables, derived from BioClim and ISRIC, are: Total available soil water content, BIO12 = mean annual precipitation, BIO15 = precipitation seasonality, bIO16 = precipitation of the wettest quarter, BIO17 = precipitation of the driest quarter, BIO18 = precipitation of the warmest quarter, BIO19 = precipitation of the coldest quarter. Total available soil water content, BIO12, and BIO15 were maintained in the multiple regression model.
(TIF)

S1 Table P values for correlation matrix of seven bioclimactic variables related to preciptation. TotalAvailSoilWater = total available soil water capacity, BIO12 = annual mean precipitation, BIO15 = precipitation seasonality, BIO16 = prrecipitation of the wettest quarter, BIO17 = preciptation of the driest quarter, BIO18 = precipitation of the warmest quarter, BIO19 = precipitation of the coldest quarter.
(DOCX)

S2 Table Mean separation for plant biomass and height under two irrigation treatments. (DOCX)

S3 Table Estimated marginal means of three traits with significant interactions for accession and irrigation from a greenhouse soil water deficit experiment on chile pepper (*Capsicum* sp.) at the Ohio State University. Organized by country of origin and alphabetically.
(DOCX)

S4 Table Multivariate regression analysis for five traits with environmental parameters related to precipitation from a greenhouse soil water deficit experiment on chile pepper (*Capsicum* sp.) at the Ohio State University. (DOCX)

S1 File (R)

S1 Data (CSV)

S2 Data (CSV)
