# Supplementary figures and images for: Environment of origin and domestication affect morphological, physiological, and agronomic response to water deficit in chile pepper (Capsicum sp.)
Source: PLoS One. 2022 Jun 14;17(6):e0260684. doi: 10.1371/journal.pone.0260684 (PMC9197065; doi:10.1371/journal.pone.0260684)

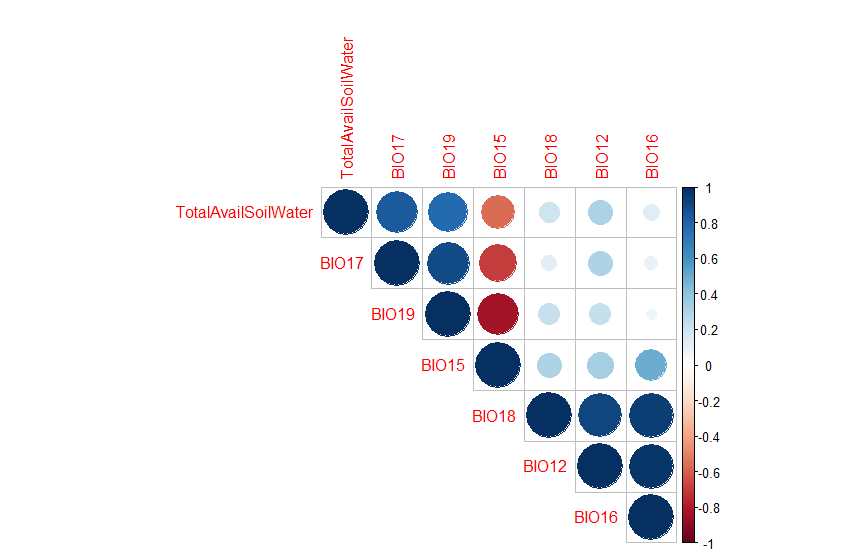

Supplement: S1 Fig — Size and color indicate strength of the corelation (larger and darger blue is more positively correlated and larger and darger red is more negatively correlated). Variables, derived from BioClim and ISRIC, are: Total available soil water content, BIO12 = mean annual precipitation, BIO15 = precipitation seasonality, bIO16 = precipitation of the wettest quarter, BIO17 = precipitation of the driest quarter, BIO18 = precipitation of the warmest quarter, BIO19 = precipitation of the coldest quarter. Total available soil water content, BIO12, and BIO15 were maintained in the multiple regression model. (TIF) [file pone.0260684.s001.tif]
