# Supplementary material for: Environment of origin and domestication affect morphological, physiological, and agronomic response to water deficit in chile pepper (Capsicum sp.)
Source: PLoS One. 2022 Jun 14;17(6):e0260684. doi: 10.1371/journal.pone.0260684 (PMC9197065; doi:10.1371/journal.pone.0260684)
Supplement: S1 Table — TotalAvailSoilWater = total available soil water capacity, BIO12 = annual mean precipitation, BIO15 = precipitation seasonality, BIO16 = prrecipitation of the wettest quarter, BIO17 = preciptation of the driest quarter, BIO18 = precipitation of the warmest quarter, BIO19 = precipitation of the coldest quarter. (DOCX) [file pone.0260684.s002.docx]

| **Table S1.** P values for correlation matrix of seven bioclimactic variables related to preciptation. TotalAvailSoilWater = total available soil water capacity, BIO12 = annual mean precipitation, BIO15 = precipitation seasonality, BIO16 = prrecipitation of the wettest quarter, BIO17 = preciptation of the driest quarter, BIO18 = precipitation of the warmest quarter, BIO19 = precipitation of the coldest quarter. | | | | | | | |
| --- | --- | --- | --- | --- | --- | --- | --- |
|  | **TotalAvailSoilWater** | **BIO12** | **BIO15** | **BIO16** | **BIO17** | **BIO18** | **BIO19** |
| **TotalAvailSoilWater** | NA | 0.498 | 0.191 | 0.780 | 0.020 | 0.664 | 0.040 |
| **BIO12** | 0.498 | NA | 0.461 | 0.000 | 0.499 | 0.004 | 0.618 |
| **BIO15** | 0.191 | 0.461 | NA | 0.262 | 0.080 | 0.506 | 0.021 |
| **BIO16** | 0.780 | 0.000 | 0.262 | NA | 0.844 | 0.001 | 0.897 |
| **BIO17** | 0.020 | 0.499 | 0.080 | 0.844 | NA | 0.796 | 0.006 |
| **BIO18** | 0.664 | 0.004 | 0.506 | 0.001 | 0.796 | NA | 0.624 |
| **BIO19** | 0.040 | 0.618 | 0.021 | 0.897 | 0.006 | 0.624 | NA |
