# Supplementary material for: Environment of origin and domestication affect morphological, physiological, and agronomic response to water deficit in chile pepper (Capsicum sp.)
Source: PLoS One. 2022 Jun 14;17(6):e0260684. doi: 10.1371/journal.pone.0260684 (PMC9197065; doi:10.1371/journal.pone.0260684)
Supplement: S2 Table — (DOCX) [file pone.0260684.s003.docx]

| **Table S2.** Mean separation for plant biomass and height under two irrigation treatments. | | | | |
| --- | --- | --- | --- | --- |
| **Irrigation** | **Plant biomass (g)^a^** | **SE^b^** | **Plant height (cm)** | **SE** |
| Control | 64.70a | 0.93 | 61.40a | 2.1 |
| Drought | 54.1b | 0.93 | 53. 80b | 2.1 |
| ^a^Different letters indicate significant differences (P = 0.05) between levels of irrigation. | | | |  |
| ^b^Indicates standard error of the mean. | | | |  |
