# Supplementary material for: Environment of origin and domestication affect morphological, physiological, and agronomic response to water deficit in chile pepper (Capsicum sp.)
Source: PLoS One. 2022 Jun 14;17(6):e0260684. doi: 10.1371/journal.pone.0260684 (PMC9197065; doi:10.1371/journal.pone.0260684)
Supplement: S3 Table — Organized by country of origin and alphabetically. (DOCX) [file pone.0260684.s004.docx]

| **Table S3.** Estimated marginal means of three traits with significant interactions for accession and irrigation from a greenhouse soil water deficit experiment on chile pepper (*Capsicum* sp.) at the Ohio State University. Organized by country of origin and alphabetically. | | | | | | | |  |
| --- | --- | --- | --- | --- | --- | --- | --- | --- |
| **Accession** | **Irrigation^a^** | **Primary Branching^b^** | **SE^c^** | **CO_2_ Assimilation (µmol/m^2^)^d^** | **SE** | **Fruit Weight (g)^e^** | **SE** |  |
|  |  |  |  |  |  |  |  |  |
| U.S. Germplasm | | | | | | | |  |
| Anaheim M | Control | 3.67 | 0.65 | 0.63 | 0.57 | 26.10 | 2.42 |  |
|  | Water Deficit | 3.67 | 0.65 | 0.22 | 0.46 | 5.57 | 2.42 |  |
| Anaheim TMR23 | Control | 2.33 | 0.65 | 1.26 | 0.57 | 21.27 | 2.42 |  |
|  | Water Deficit | 2.67 | 0.65 | 1.03 | 0.46 | 8.06 | 2.42 |  |
| Canoncito | Control | 2.67 | 0.65 | 0.57 | 0.46 | 15.70 | 2.42 |  |
|  | Water Deficit | 2.33 | 0.65 | 1.32 | 0.46 | 14.15 | 2.42 |  |
| Chilhuacle Negro | Control | 6.67 | 0.65 | -1.03 | 0.46 | 12.34 | 2.42 |  |
|  | Water Deficit | 3.67 | 0.65 | -0.43 | 0.57 | 6.88 | 2.42 |  |
| Chimayo | Control | 3.00 | 0.65 | -0.61 | 0.46 | 15.44 | 2.42 |  |
|  | Water Deficit | 2.67 | 0.65 | -0.21 | 0.46 | 8.21 | 2.42 |  |
| Hidalgo Hot | Control | 5.33 | 0.65 | 0.96 | 0.46 | 3.91 | 2.42 |  |
|  | Water Deficit | 5.00 | 0.65 | 1.63 | 0.46 | 1.83 | 2.42 |  |
| Kaala | Control | 6.67 | 0.65 | 1.57 | 0.46 | 0.12 | 2.42 |  |
|  | Water Deficit | 3.00 | 0.65 | 1.69 | 0.46 | 0.00 | 2.42 |  |
| NuMex Conquistador | Control | 2.67 | 0.65 | 0.44 | 0.46 | 21.71 | 2.42 |  |
|  | Water Deficit | 3.00 | 0.65 | 1.69 | 0.46 | 12.02 | 2.42 |  |
| PI159229 | Control | 7.67 | 0.65 | 0.71 | 0.46 | 8.58 | 2.42 |  |
|  | Water Deficit | 5.67 | 0.65 | 0.95 | 0.57 | 2.04 | 2.42 |  |
| PI586665 | Control | 4.00 | 0.65 | 0.80 | 0.46 | 13.50 | 2.42 |  |
|  | Water Deficit | 3.00 | 0.65 | -0.64 | 0.46 | 5.25 | 2.42 |  |
| PI586666 | Control | 3.00 | 0.65 | 0.92 | 0.46 | 17.72 | 2.42 |  |
|  | Water Deficit | 3.00 | 0.65 | -1.17 | 0.46 | 2.41 | 2.42 |  |
| PI592813 | Control | 3.00 | 0.65 | 0.72 | 0.46 | 19.39 | 2.42 |  |
|  | Water Deficit | 2.33 | 0.65 | 1.01 | 0.46 | 6.24 | 2.42 |  |
| PI631153 | Control | 8.00 | 0.65 | 0.85 | 0.46 | 5.67 | 2.42 |  |
|  | Water Deficit | 10.67 | 0.65 | 0.80 | 0.46 | 2.99 | 2.42 |  |
| Stocky Golden Roaster | Control | 2.33 | 0.65 | 0.35 | 0.46 | 20.16 | 2.42 |  |
|  | Water Deficit | 2.33 | 0.65 | 0.13 | 0.81 | 1.43 | 2.42 |  |
| Szegedi 179 | Control | 4.33 | 0.65 | 1.75 | 0.57 | 22.03 | 2.42 |  |
|  | Water Deficit | 3.33 | 0.65 | -0.69 | 0.46 | 12.36 | 2.42 |  |
| Tam Jalapeno | Control | 3.00 | 0.65 | 0.63 | 0.46 | 7.15 | 2.42 |  |
|  | Water Deficit | 3.00 | 0.65 | 0.68 | 0.46 | 3.58 | 2.42 |  |
| Tam Vera Cruz | Control | 2.00 | 0.65 | 1.13 | 0.46 | 5.96 | 2.42 |  |
|  | Water Deficit | 2.33 | 0.65 | 1.66 | 0.46 | 2.66 | 2.42 |  |
| Waialua | Control | 4.67 | 0.65 | 1.68 | 0.46 | 7.65 | 2.42 |  |
|  | Water Deficit | 2.67 | 0.65 | 0.89 | 0.46 | 1.24 | 2.42 |  |
| Mexico Germplasm | | | | | | | |  |
| Ca0045 | Control | 11.67 | 0.65 | -0.97 | 0.57 | NA | NA |  |
|  | Water Deficit | 11.00 | 0.65 | 0.03 | 0.57 | NA | NA |  |
| Ca0057 | Control | 5.33 | 0.65 | 0.78 | 0.46 | NA | NA |  |
|  | Water Deficit | 5.00 | 0.65 | 0.70 | 0.46 | NA | NA |  |
| Ca0256 | Control | 11.00 | 0.65 | 1.26 | 0.46 | NA | NA |  |
|  | Water Deficit | 7.33 | 0.65 | 1.17 | 0.46 | NA | NA |  |
| Ca0310 | Control | 7.00 | 0.65 | 0.85 | 0.46 | NA | NA |  |
|  | Water Deficit | 5.00 | 0.65 | 1.58 | 0.46 | NA | NA |  |
| Ca0344 | Control | 3.00 | 0.65 | 0.56 | 0.46 | NA | NA |  |
|  | Water Deficit | 3.67 | 0.65 | 1.29 | 0.46 | NA | NA |  |
| Cc0144 | Control | 2.00 | 0.65 | -0.76 | 0.46 | NA | NA |  |
|  | Water Deficit | 2.67 | 0.65 | 0.38 | 0.46 | NA | NA |  |
| Cf0173 | Control | 8.33 | 0.65 | 1.10 | 0.46 | NA | NA |  |
|  | Water Deficit | 8.00 | 0.65 | 1.08 | 0.46 | NA | NA |  |
| ^a^Irrigation treatment has two levels: Control = daily watering, Water Deficit = weekly watering. | | | | | | | |  |
| ^b^Mean number of primary branches. | | | | | | | |  |
| ^c^Indicates standard error of the mean. | | | | | | | |  |
| ^d^Mean net CO_2_ assimilation collected with the LI-6800 (LICOR Biosciences, Lincoln, NE). | | | | | | | |  |
| ^e^Mean dried fruit weight. Collected only in US accessions due to differing phenology between US and Mexico accessions. | | | | | | | |  |
