# Supplementary material for: Environment of origin and domestication affect morphological, physiological, and agronomic response to water deficit in chile pepper (Capsicum sp.)
Source: PLoS One. 2022 Jun 14;17(6):e0260684. doi: 10.1371/journal.pone.0260684 (PMC9197065; doi:10.1371/journal.pone.0260684)
Supplement: S4 Table — (DOCX) [file pone.0260684.s005.docx]

| **Table S4.** Multivariate regression analysis for five traits with environmental parameters related to precipitation from a greenhouse soil water deficit experiment on chile pepper (*Capsicum* sp.) at the Ohio State University. | | | | | |
| --- | --- | --- | --- | --- | --- |
| **Trait** | **Coefficients** | **Estimate^a^** | **SE^b^** | **R^2^** | **P^c^** |
| CO_2_ Assimilation | Irrigation | -16.270 | 41.050 | -0.009 | NS |
|  | Total Available Soil Water | -0.269 | 0.534 |  | NS |
|  | Annual Mean Temperature | -0.002 | 0.003 |  | NS |
|  | Precipitation Seasonality | -0.082 | 0.088 |  | NS |
|  | Irrigation by Total Available Soil Water | 0.360 | 0.756 |  | NS |
|  | Irrigation by Annual Mean Precipitation | 0.001 | 0.004 |  | NS |
|  | Irrigation by Precipitation Seasonality | 0.008 | 0.125 |  | NS |
| Stomatal Conductance | Irrigation | 1.855 | 2.344 | -0.127 | NS |
|  | Total Available Soil Water | -0.005 | 0.029 |  | NS |
|  | Annual Mean Temperature | 0.000 | 0.000 |  | NS |
|  | Precipitation Seasonality | 0.000 | 0.005 |  | NS |
|  | Irrigation by Total Available Soil Water | -0.031 | 0.041 |  | NS |
|  | Irrigation by Annual Mean Precipitation | 0.000 | 0.000 |  | NS |
|  | Irrigation by Precipitation Seasonality | -0.006 | 0.008 |  | NS |
| Plant Biomass | Irrigation | 4.637 | 210.703 | 0.311 | NS |
|  | Total Available Soil Water | 3.935 | 2.711 |  | NS |
|  | Annual Mean Temperature | -0.008 | 0.014 |  | NS |
|  | Precipitation Seasonality | -0.125 | 0.461 |  | NS |
|  | Irrigation by Total Available Soil Water | -0.838 | 3.834 |  | NS |
|  | Irrigation by Annual Mean Precipitation | -0.016 | 0.020 |  | NS |
|  | Irrigation by Precipitation Seasonality | 0.336 | 0.652 |  | NS |
| Plant Height | Irrigation | 3.860 | 195.112 | 0.199 | NS |
|  | Total Available Soil Water | -3.755 | 2.510 |  | NS |
|  | Annual Mean Temperature | -0.006 | 0.013 |  | NS |
|  | Precipitation Seasonality | -0.630 | 0.427 |  | NS |
|  | Irrigation by Total Available Soil Water | 0.043 | 3.550 |  | NS |
|  | Irrigation by Annual Mean Precipitation | 0.007 | 0.019 |  | NS |
|  | Irrigation by Precipitation Seasonality | -0.192 | 0.604 |  | NS |
| \| ^a^Estimate indicates the change in the trait relative to one unit change of the coefficient. \| \| \| --- \| --- \| \| ^b^Indicates standard error of the mean. \|  \| \| ^c^*, **, ***specify significant relationship at P values of 0.05, 0.01, and 0.001 respectively. \| \| | | | | | |
